# Mesenchymal stem cells from human amniotic membrane differentiate into cardiomyocytes and endothelial-like cells without improving cardiac function after surgical administration in rat model of chronic heart failure

**DOI:** 10.15171/jcvtr.2019.06

**Published:** 2019-02-25

**Authors:** Fazel Gorjipour, Ladan Hosseini-Gohari, Alireza Alizadeh Ghavidel, Seyed Javad Hajimiresmaiel, Nasim Naderi, Amir Darbandi Azar, Hamidreza Pazoki-Toroudi

**Affiliations:** ^1^Cellular and Molecular Research center, Iran University of Medical Sciences, Tehran, Iran; ^2^Rajaie Cardiovascular and Medical and Research Center, Iran University of Medical Sciences, Tehran, Iran; ^3^Department of Cardiology, Faculty of Medicine, Iran University of Medical Sciences, Tehran, Iran; ^4^Department of Physiology and Physiology Research Center, Faculty of Medicine, Iran University of Medical Sciences, Tehran, Iran

**Keywords:** Mesenchymal Stromal Cells, Chronic Heart Failure, Amnion

## Abstract

***Introduction:*** Human amnion-derived mesenchymal stem cells (hAMSCs) have been used in the treatment of acute myocardial infarction. In the current study, we investigated the efficacy of hAMSCs for the treatment of chronic model of myocardial ischemia and heart failure (HF) in rats.

***Methods:*** Male Wistar rats weighing between 250 to 350 g were randomized into three groups: sham, HF control and HF+hAMSCs. For HF induction, animals were anesthetized and underwent left anterior descending artery ligation. In HF+hAMSCs group, 2×106 cells were injected into the left ventricular muscle four weeks post ischemia in the border zone of the ischemic area. Cardiac function was studied using echocardiography. Masson’s trichrome staining was used for studying tissue fibrosis. Cells were transduced with green fluorescent protein (GFP) coding lentiviral vector. Immunohistochemistry was used for detecting GFP, vascular-endothelial growth factor (VEGF) and troponin T markers in the tissue sections.

***Results:*** Assessment of the cardiac function revealed no improvement in the myocardial function compared to the control HF group. Moreover, tissue fibrosis was similar in two groups. Immunohistochemical study revealed the homing of the injected hAMSCs to the myocardium. Cells were stained positive for VEGF and troponin T markers.

***Conclusion:*** injection of hAMSCs 4 weeks after ischemia does not improve cardiac function and cardiac muscle fibrosis, although the cells show markers of differentiation into vascular endothelial cells and cardiomyocytes. In sum, it appears that hAMSCs are effective in the early phases of myocardial ischemia and does not offer a significant advantage in patients with chronic HF.

## Introduction

Heart failure (HF) is among the main causes of mortality and morbidity. Patients with HF following chronic ischemic heart diseases usually are presented with poor prognosis. Current treatment modalities are mostly palliative and are aimed at alleviating the symptoms and controlling the advancement of the disease.^1-4^ Final therapeutic option is organ transplantation, which is seriously impeded by donor shortage, donor matching and complication rising during and after cardiac transplantation procedure.^2,5,6^ The operation is very risky and patients need to take immunosuppressive drugs for their whole life, although despite lifelong immunosuppressive therapy, there is a possibility for transplant rejection.^7-9^ Therefore, search for new therapeutic options is a must for treating patients with HF. One of the recent approaches which stands a chance for being used for the treatment of the disease is cellular cardiomyoplasty, use of stem cells to repair regions of damaged or necrotic myocardium.^10^ Cell-based approaches have shown promise in some studies for the treatment of acute and subacute myocardial ischemia. Mesenchymal stem cells (MSCs) have gained popularity for their use in myocardial regeneration.^11^ According to the International Society for Cellular Therapy (ISCT) minimal criteria for defining the multipotent MSCs, these are plastic-adherent cells, expressing CD105, CD73 and CD90 markers and lacking the surface presentation of CD45, CD34, CD14 or CD11b, CD79a or CD19 and HLA-DR markers. Moreover, they have the capacity for in vitro differentiation to osteoblasts, adipocytes and chondroblasts.^12^ These cells could be obtained from various sources in the body, and despite common features defined by the minimal criteria set of the ISCT, have some differences in their replicative capacity and therapeutic potential. One of these sources is the fetal membranes, including amniotic membrane.^13^ The amniotic membrane and other fetal adnexa are regarded as wastes of childbirth and as a source of stem cells, they are specially interesting due to the availability without bearing comorbidity to the donor.^13^ Recent studies have shown the promise of these cells in the treatment of acute and subacute myocardial ischemia and ameliorated HF in the rodent models of these disease.^13-15^ However, it is not clearly defined if treatment with these cells may offer any benefits in patients suffering from chronic ischemic HF. Although the MSCs are shown to differentiate to cells with myocardial and endothelial features, doubts exist regarding their functionality, and even some studies have suggested that myocardial cells differentiated from MSCs lack the features of a viable cardiomyocyte with contractile function. Most of the studies suggest that MSCs are effective in acute myocardial ischemia due to their immunomodulatory properties as well as proangiogenic properties. Actually, during acute ischemia there are elevated oxidative stress and inflammatory response, which cause the cell death of the ischemic tissue.^16-24^ These cells help improve tissue hemostasis through modulating the immune response and improving blood flow by angiogenesis induction and sparing the existing cardiomyocytes from apoptotic and necrotic cell death.^11,13,14,25^ Therefore, studying their applicability in chronic ischemic HF, where severe inflammatory response and myocardial cell death have led to tissue remodeling and tissue fibrosis, is required.

In the current study, we hypothesized that MSC from human term amniotic membrane might differentiate into cardiomyocyte or endothelial-like cells and improve the myocardial function in the chronic ischemic HF rat model when administered surgically. The potential of these cells for homing in the myocardial tissue of the rat model of HF following surgical intramyocardial injection, altering cardiac function, regenerating fibrotic tissue, and their potential in vivo differentiation to the cardiomyocytes and endothelial-like cells are investigated.

## Materials and Methods

### Cell isolation and characterization

Cells were isolated and characterized as previously described.^25^ Human placentas were obtained from healthy female donors during caesarean section with prior informed consent of the donors. After transfer to the laboratory in sterile phosphate-buffered saline (PBS) and antibiotics, the amniotic membrane was isolated from placenta and cut into small pieces. Cells were dissociated using collagenase type I (Gibco, Grand Island, USA) in a shaking incubator at 37°C for 1–2 hours. Then, cells were suspended in the low glucose DMEM culture medium (Sigma Aldrich, Gillingham, Wisconsin, USA) containing 10% FBS (Invitrogen, Camarillo, California, USA) and 1% of standard Penicillin-Streptomycin solution (10 000 units penicillin and 10 mg streptomycin/ml) (Gibco, Grand Island, USA). Then, cells were seeded into T75 flasks (Nunc, Roskilde, Denmark). The flasks were placed in an incubator at 37°C in 5% CO2 and 95% humidity. After three passages, cells were tested for the following cell surface markers: CD34-PE, CD44-FITC, CD45-FITC, CD73-PE, CD90-PE and CD105-FITC by flow cytometry technique (Partec 3, USA). Osteoblast and adipocyte differentiation capability of hAM-MSCs was tested in vitro as previously described.^26^

### Lentivirus-green fluorescent protein (Lenti-GFP) transduction for tracking

Gene delivery to the hAMSCs was performed using a second generation lentiviral vector system.^27^ pLV-IRES-GFP, a bicistronic lentiviral vector with an EF-1 alpha promoter was used for the transduction of the cells, their labeling with GFP and tracking of the transplanted cells in vivo using GFP fluorescent microscopy of the tissue sections. The process for preparation and viral transduction of the cells has been described elsewhere.^26^

### Animal experiments and hAMSCs administration

Twenty-four male Wistar rats weighing between 250 to 350 g were provided by Laboratory Animal, Breeding and Husbandry Center, Iran University of Medical Sciences, Tehran, Iran. All animals were stored under standard conditions in cages at a controlled temperature (25°C), with daily exposure to a 20-hour light-dark cycle and free access to standard laboratory food and tap water. Animals were randomly allocated to three groups: Sham group received only chest opening without any extra manipulation; HF control group underwent left anterior descending artery (LAD) ligation and received PBS as the carrier 4 weeks later and HF+hAMSCs group, which underwent LAD ligation and received hAMSCs (passage 6, P6) as treatment four weeks later.

LAD ligation was performed using a 6-0 Prolene (Ethicon Inc., USA) suture as previously described.^10,28,29^ Animals were anesthetized by intra-peritoneal administration of a mixture of 75 mg/kg ketamine and 5 mg/kg xylazine. Then, animals were orally intubated with a 16-gauge intravenous catheter and placed in the supine position on a warming pad with anal temperature control thermometer. Animals were ventilated with the room air using a modified infant ventilator (Sechrist Industries, USA) during operation. Electrocardio-graphic monitoring was applied for monitoring the vital signs. The heart was exposed via left thoracotomy by cutting the fourth and fifth ribs. The pericardial sac was opened, and LAD was ligated permanently. Pale discoloration of the left ventricular muscle was regarded as the confirmation of the successful ligation of the LAD. To stitch the muscle layer and skin incision, 4-0 Prolene and 2-0 silk suture were respectively used. At the end of surgery, erythromycin (10 mg/kg, SC), ketoprofen 10% (10 mg/kg IM.), and warm sterile saline (1-2 mL, SC) were injected, and the rats were left on the heating pad until they had recovered from anesthesia. After that, rats were extubated and removed from the ventilator. The dose of flunixin was repeated every 6 to 12 hours.

Cell administration was done four weeks following LAD ligation in four points around the ischemic area. 2×10^6^ cells were suspended in 200 µL of PBS and were immediately injected into the myocardium of the left ventricle in the border zone of the ischemic area.

For assessing the myocardial function, rats underwent ketamine-xylazine anesthesia for echocardiographic examination of the heart. A Vivid 7 echocardiography machine (GE Healthcare Italia, Milano, Italy) equipped with 10 MHz phased array transducer was utilized for this purpose as previously described.^30^ Ejection fraction(EF) and fractional shortening (FS) were acquired and calculated for each animal.

### Tissue staining and immunohistochemistry

About 4 weeks after stem cell administration or serving as either sham or untreated control or vehicle, animals were sacrificed and their hearts were isolated and embedded in paraffin after fixation with 10% paraformaldehyde solution. Three sections (5 μm thick) were prepared with a 2000-μm interval from apex for trichrome staining or immunohistochemical analysis.

Masson’s trichrome staining was performed on cardiac sections to estimate the total amount of damage and fibrosis of the myocardial tissue as previously described.^31^

Immunohistochemistry was used to detect the expression of GFP, vascular-endothelial growth factor (VEGF) or cardiac troponin T in the myocardial tissue. For studying the endothelial- or cardiomyocyte differentiation of the introduced hAMSCs, tissue sections for double stained for the presence of GFP and either VEGF or troponin T. For this purpose, the tissue sections were incubated for 45 minutes at 37°C in a blocking solution (10% normal goat serum, 3% Triton X-100 in PBS) followed by incubation in a monoclonal anti-GFP (orb10709, Biorbyt, Cambridge, UK), anti-VEGF (orb11554, Biorbyt, Cambridge, UK) or anti-troponin T (orb182936, Biorbyt, Cambridge, UK) antibodies in PBS for overnight at 4°C. Then, sections washed in PBS (3×5 minutes) and incubated with respective secondary antibody in PBS for 2 hours at room temperature. DAPI solution (1 mg/mL of 4′,6-diamidino-2-phenylindole) incubation for nuclear DNA staining was performed for 30 seconds in the dark. The amount of GFP protein expression (green) and VEGF or troponin T were analyzed using a fluorescent microscope (Olympus Corporation, Japan) with higher magnifications (400×) and counted on five random fields for each section by a blinded investigator to experimental design. Data was analyzed by ImageJ software and presented as double positive cell count percent to cells positive for GFP.

### Statistical analysis

Statistical analysis was done using SPSS software version 18 (IBM Incorporation, New York, USA). One-way analysis of variance (ANOVA) test was used for comparison of parameters between three groups and post-hoc Mann-Whitney U test was done for pairwise comparison of the groups. Mixed ANOVA with Bonferroni’s adjustment was used to compare repeated measures of outcomes whenever required. The power of analysis was 80%, and the level of significance was 0.05.

## Results

**Figure 1:**
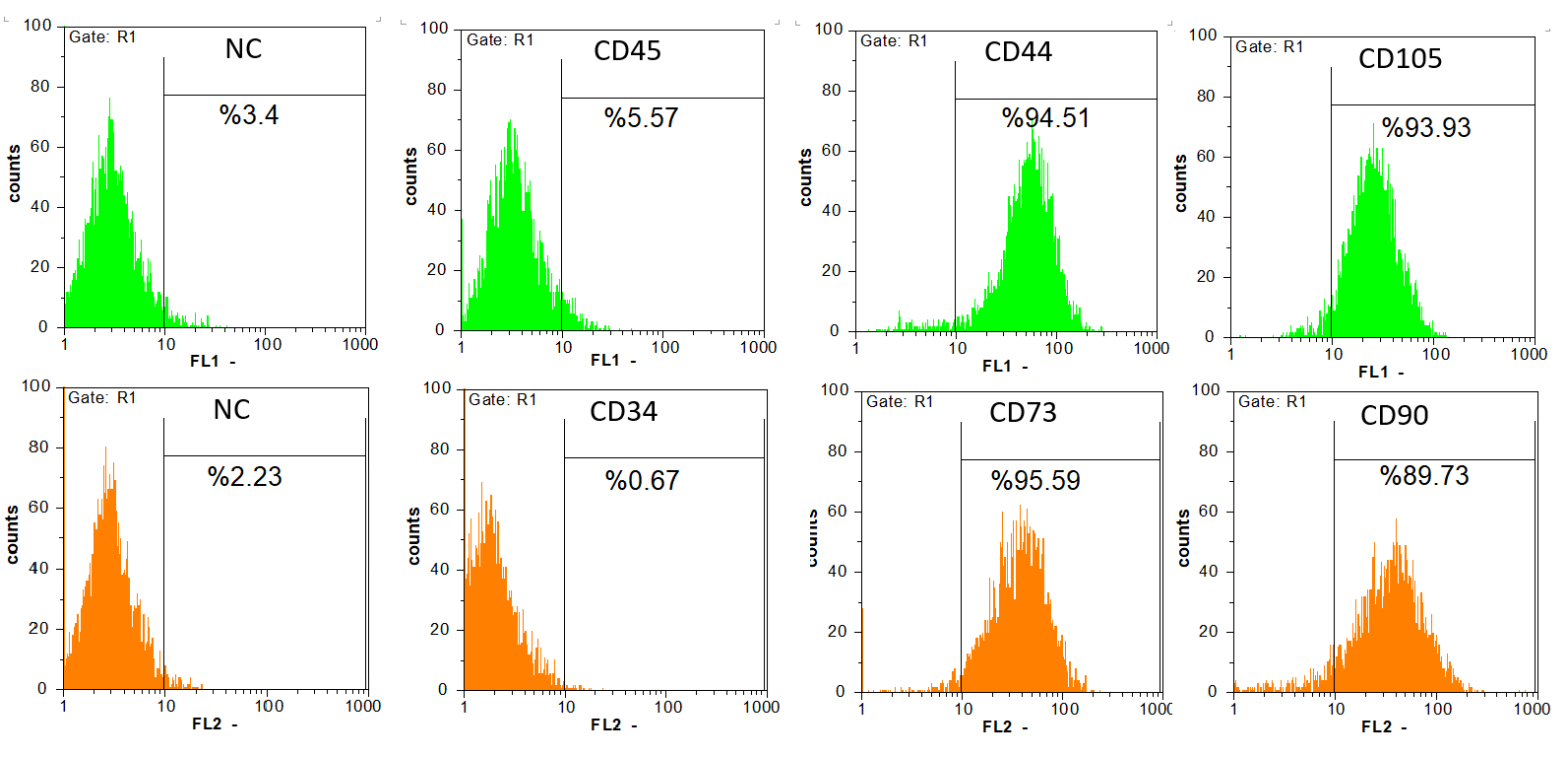
Immunophenotyping of the human amniotic membrane derived mesenchymal stem cells (hAMSCs) for their surface CD markers using flowcytometry confirmed their lineage. CD: cluster of differentiation, NC: negative control (isotype control).

**Figure 2:**
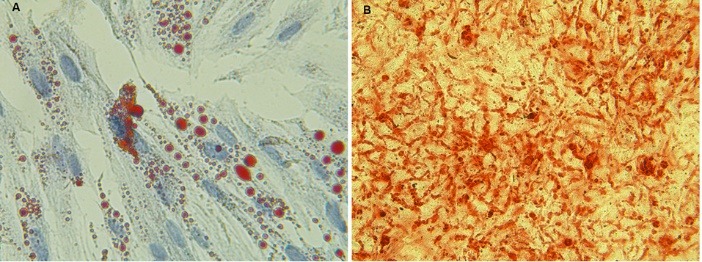
Oil red and alizarin red staining for differentiation of the hAMSCs to adipocyte (A) and osteocyte (B), respectively, demonstrated the potency of the cells to differentiate into these two cell lineages. (A) The fat droplets within the adipocytes differentiated from hAMSCs appear reddish-orange after Oil Red O stain (magnification 400X). (B) The calcified nodules appeared bright red in color after alizarin red staining of the osteocyte-differentiated hAMSCs (magnification ×100).

MSCs were defined with their spindle-form shape and adherent phenotype on culture plates during cell culture. Further characterization for confirming the identity of the cells demonstrated the positivity of the cell populations for CD44, CD73, CD90 and CD105 markers and negativity for CD34 and CD45 markers by flow cytometry technique (Figure 1). These as well as the potency of the cells to differentiate into the osteoblasts and adipocytes confirmed their identity as stem cells of mesenchymal type (Figure 2).

**Figure 3:**
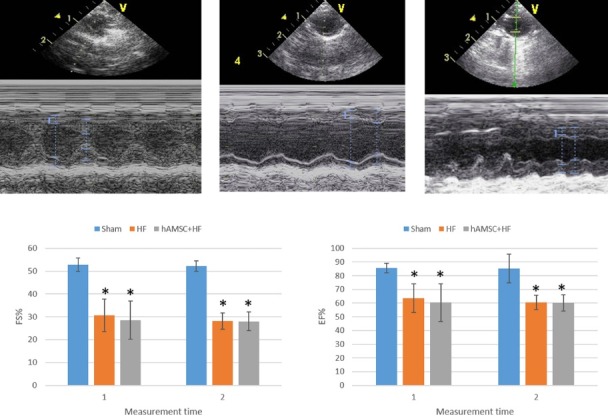
Echocardiographic data with comparisons between groups over a period of 4 weeks ­­following human amniotic membrane-derived mesenchymal stem cells (hAMSCs) administration. Sham: sham-operated group, HF: heart failure, hAMSCs: human amniotic mesenchymal stem cells. Upper row from left to right: representative figures of Sham, HF and HF+hAMSCs groups echocardiographic study; lower row: comparison of fractional shortening (FS%) and ejection fraction (EF%) between three groups. Measurements were performed at time 1, immediately before control intervention or cell administration and time 2, four weeks following the first measurement. * *P*<0.05 against Sham group.

**Figure 4:**
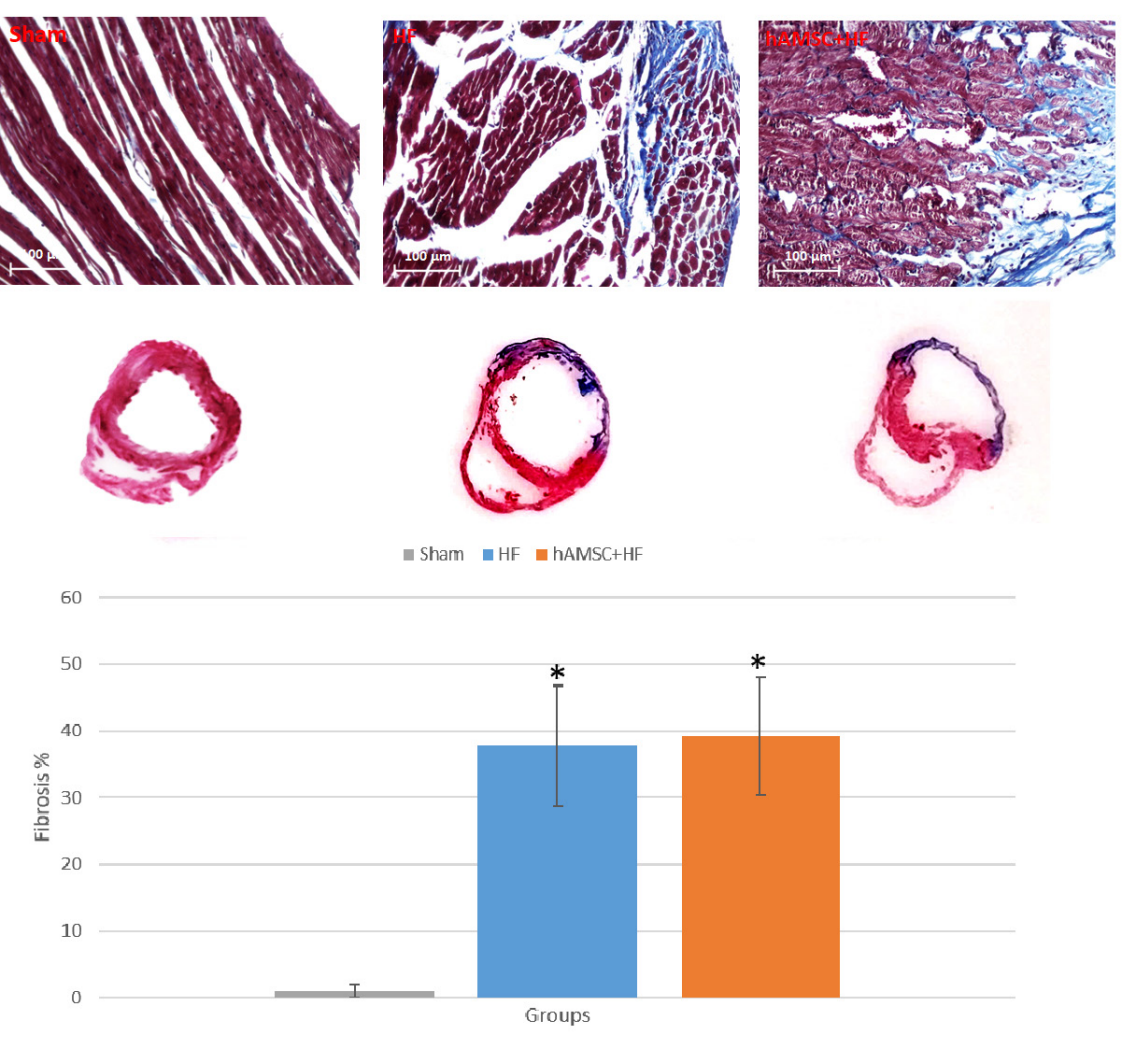
Tissue staining with Masson’s trichrome method with comparisons between groups over a period of 4 weeks following human amniotic membrane-derived mesenchymal stem cells (hAMSCs) administration. Left panel demonstrates a sample from animals in the untreated patients and the right one is depicting the treated animal. * *P* < 0.05 against Sham group.

After administration of the cells to the left ventricular myocardium, the cardiac function was measured on several occasions (before and four weeks after the cell administration) to see whether surgical treatment with hAMSCs can improve HF and/or prevent further loss of cardiac function. According to the results (Figure 3) echocardiographic measures demonstrated that both HF control and treatment groups had significant decrease in their EF and fractional shortening (*P *< 0.01) compared with the sham group. The findings are consistent with the characteristics of the dilated cardiomyopathy. However, significant differences between 2 groups, the cell therapy group compared with the untreated control group, were not observed in case of either EF and FS (*P *> 0.05). Histological staining with trichrome dyes and following quantification of the injured tissue demonstrated similar trend in the fibrosis of the myocardial tissue and the deposition of collagen (Figure 4). A severe left ventricular fibrosis along with dilatation of the left ventricle was observed in both groups in comparison with the sham group. However, the outcomes were similar between cell therapy and control groups.

**Figure 5:**
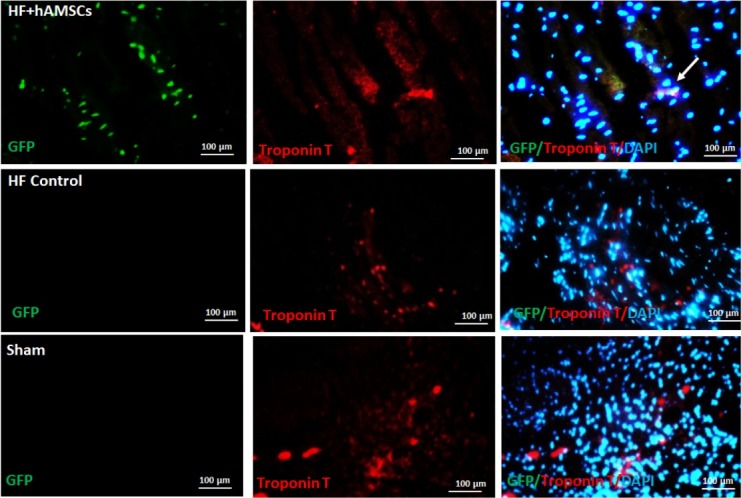
Immunofluorescence analysis of the samples from animals transplanted with human amniotic membrane-derived mesenchymal stem cells (hAMSCs) in their left ventricle demonstrate features of cell homing in the injured tissue as wells as their differentiation capability to cardiomyocyte-like cells. Arrow shows the double positive cells for GFP and cardiac troponin T (cTnT). GFP: green fluorescent protein, DAPI:. 4′,6-diamidino-2-phenylindole.

**Figure 6:**
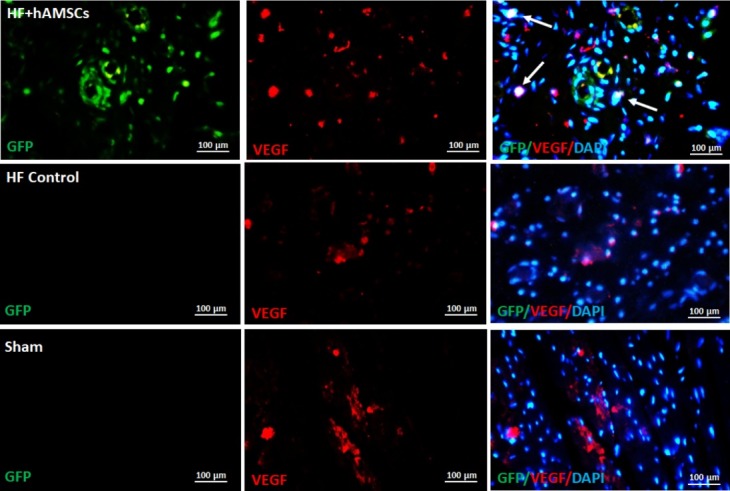
Immunofluorescence analysis of the samples from animals transplanted with human amniotic membrane-derived mesenchymal stem cells (hAMSCs) in their left ventricle demonstrate features of cell homing in the injured tissue as wells as their differentiation capability to endothelial-like cells. Arrows show the double positive cells for GFP and vascular endothelial growth factor (VEGF). GFP: green fluorescent protein, VEGF: vascular endothelial growth factor, DAPI: 4′,6-diamidino-2-phenylindole.

Further follow-up immunohistochemical staining for GFP demonstrated that cells are homing in the myocardial tissue in the injured area. The cells homing at the myocardium demonstrated the expression of molecular markers of the cardiomyocytes (cardiac troponin T) (Figure 5) and endothelial cells (VEGF) (Figure 6) as obtained by double staining for GFP and either of the mentioned markers. This demonstrates that hAMSCs show some phenotypic features of the cardiomyocytes or endothelial cells and have the potential for cardiac regeneration.

## Discussion

MSCs have been used for the treatment of acute myocardial infarction models.^10,13-15^ The cells have been isolated from various sources, including allogeneic and autologous donors and tissue origins such as bone marrow, adipose tissue and childbirth wastes.^12^ MSCs from amniotic membrane have been tested for the treatment of acute myocardial infarction and HF related to coronary heart disease. However, most interventions have used acute myocardial ischemia model and investigated its effectiveness in the treatment of MI immediately or shortly after it.^10,15^ While results from these studies prove the preclinical efficacy of this type of the cells in the treatment of MI, it is not clear whether these cells are effective in the treatment of chronic MI associated with severe impairment of the heart function, advanced ventricular dilatation and HF. In the current study using rat models of HF related to chronic myocardial ischemia, we demonstrated that hAMSCs may not be effective in improving heart function or maintaining its function over the time. Indeed, treatment with hAMSCs did not show any effects on the HF and cardiac function parameters in the current study. Animals treated with this type of the cells were similar to untreated control groups in terms of ejection fraction and fractional shortening. Further histological examination demonstrated that the animals had a very similar amount of tissue fibrosis and myocardial collagen deposition. Contrary to our findings, Tsuji et al have found that xenograft of hAMSCs in subacute model of myocardial ischemia, when administered as early as two weeks following ischemia, in rats improved cardiac function.^32^ hAMSCs are mostly effective in the early phases of the MI, mostly due to their immunomodulatory function, proangiogenic properties and cardiomyocyte differentiation.^32,33^ Indeed, these cells are mostly effective while early intervention could spare the cardiomyocytes from death due to metabolic disturbances and inflammatory response.^32^ While in the late phases, the tissue has lost most of its cardiomyocytes and the myocardium is broadly replaced by nonfunctional fibrotic tissue, immune modulation and angiogenesis does not offer much benefit, for the improvement of the left ventricular ejection fraction. An evidence for substantiation of this hypothesis comes from a clinical trial by Hare et al which tested the allogeneic and autologous MSCs in patients with chronic HF secondary to myocardial ischemia.^34^ Their findings demonstrated that MSCs are well tolerated and are safe in these patients but do not improve patients’ EF. Their findings are consistent with the results of the current study. Since in chronic ischemia and consequent HF the tissue loses a large fraction of its active cardiomyocytes, the reasonable regenerative approach is mostly dependent on the replacement of nonfunctional tissue with functional cardiomyocytes. While our study as well as previous studies have demonstrated the ability of these cells to differentiate into cells expressing cardiomyocyte markers, they do not acquire contractile activity required for mechanical activity of the myocardial tissue.^35^ Shadrin et al demonstrated that these cells lack the potential to contraction despite their potential for the response to stimuli and capability in electromagnetic coupling with neighboring rat ventricular myocytes. Many more studies have evidenced the cardiomyogenic differentiation of MSCs, including hAMSCs, in vitro and in vivo. They have also been demonstrated to express molecules that are involved in the formation of gap junctions.^32,34,35^ Although MSCs may show some molecular and phenotypic features of cardiac myocytes either through in vivo transdifferentiation or fusion with cardiomyocytes, they probably lack the potential stimuli response and contractility which are the final desired function expected from a treatment for failing myocardium. It is a cause for concern in cell therapy in chronic ischemic HF where a group of cardiomyocytes must work in concert and respond to stimuli to give rise to a functional myocardium. However, a limitation of the current study is to demonstrate the functional ability of the cells with myocardial-like or endothelial-like phenotypes to give rise to functional tissues and regeneration. Another limitation is to clarify if these phenotypes of MSCs are acquired through transdifferentiation or fusion with neighboring cells.

In sum, the study reveals that treatment of advanced chronic ischemic HF with hAMSCs does not offer much benefit in terms of myocardial function and tissue fibrosis. Cardiomyocyte like cells and vascular endothelial differentiation happens in a small fraction of cells and is not enough for functional recovery of cells. It appears that treatment of the myocardial ischemia in the early phases yields better outcomes.

## Ethical approval

The project was approved by the institutional ethics review board at Iran University of Medical Sciences (IUMS, Tehran, Iran; ethics committee approval code: IR.IUMS.REC.1393.25477). Moreover, the ethical standard for the care and use of laboratory animals of the Research Ethics Committee of IUMS was respected during all parts of the current study.

## Competing interests

All authors declare no competing financial interests exist.

## Acknowledgment

We would like to express our appreciation to Dr Naser Ahmadbeigi for donating the Lenti-GFP virus preparations for cell transfection.

## References

[1] Ramani GV, Uber PA, Mehra MR (2010). Chronic heart failure: contemporary diagnosis and management. Mayo Clin Proc.

[2] Adhyapak SM, Parachuri VR (2018). High-risk cardiac surgery as an alternative to transplant or mechanical support in patients with end-stage heart failure: A seemingly viable option. J Thorac Cardiovasc Surg.

[3] Lourenco AP, Leite-Moreira AF, Balligand JL, Bauersachs J, Dawson D, de Boer RA (2018). An integrative translational approach to study heart failure with preserved ejection fraction: a position paper from the Working Group on Myocardial Function of the European Society of Cardiology. Eur J Heart Fail.

[4] Atherton JJ, Sindone A, De Pasquale CG, Driscoll A, MacDonald PS, Hopper I (2018). National Heart Foundation of Australia and Cardiac Society of Australia and New Zealand: Australian clinical guidelines for the management of heart failure 2018. Med J Aust.

[5] Gunther A, Aaberge L, Abildgaard A, Ragnarsson A, Edvardsen T, Jakobsen J (2018). Coronary computed tomography in heart transplant patients: detection of significant stenosis and cardiac allograft vasculopathy, image quality, and radiation dose. Acta Radiol.

[6] Jennings DL, Lange N, Shullo M, Latif F, Restaino S, Topkara VK (2018). Outcomes associated with mammalian target of rapamycin (mTOR) inhibitors in heart transplant recipients: A meta-analysis. Int J Cardiol.

[7] Soderlund C, Radegran G (2015). Immunosuppressive therapies after heart transplantation--The balance between under- and over-immunosuppression. Transplant Rev (Orlando).

[8] Hullin R (2014). Heart transplantation: current practice and outlook to the future. Swiss Med Wkly.

[9] Boilson BA, Raichlin E, Park SJ, Kushwaha SS (2010). Device therapy and cardiac transplantation for end-stage heart failure. Curr Probl Cardiol.

[10] Tavakoli F, Ostad SN, Khori V, Alizadeh AM, Sadeghpour A, Darbandi Azar A (2013). Outcome improvement of cellular cardiomyoplasty using triple therapy: mesenchymal stem cell+erythropoietin+vascular endothelial growth factor. Eur J Pharmacol.

[11] Kobayashi K, Suzuki K (2018). Mesenchymal Stem/Stromal Cell-Based Therapy for Heart Failure- What Is the Best Source?. Circ J.

[12] Dominici M, Le Blanc K, Mueller I, Slaper-Cortenbach I, Marini F, Krause D (2006). Minimal criteria for defining multipotent mesenchymal stromal cells The International Society for Cellular Therapy position statement. Cytotherapy.

[13] Balbi C, Bollini S (2017). Fetal and perinatal stem cells in cardiac regeneration: Moving forward to the paracrine era. Placenta.

[14] Fang CH, Jin J, Joe JH, Song YS, So BI, Lim SM (2012). In vivo differentiation of human amniotic epithelial cells into cardiomyocyte-like cells and cell transplantation effect on myocardial infarction in rats: comparison with cord blood and adipose tissue-derived mesenchymal stem cells. Cell Transplant.

[15] Walther G, Gekas J, Bertrand OF (2009). Amniotic stem cells for cellular cardiomyoplasty: promises and premises. Catheter Cardiovasc Interv.

[16] Amani H, Habibey R, Hajmiresmail S, Latifi S, Pazoki-Toroudi H, Akhavan O (2017). Antioxidant nanomaterials in advanced diagnoses and treatments of ischemia reperfusion injuries. Journal of Materials Chemistry B.

[17] Ajami M, Davoodi SH, Habibey R, Namazi N, Soleimani M, Pazoki-Toroudi H (2013). Effect of DHA+EPA on oxidative stress and apoptosis induced by ischemia-reperfusion in rat kidneys. Fundam Clin Pharmacol.

[18] Amani H, Ajami M, Nasseri Maleki S, Pazoki-Toroudi H, Daglia M, Tsetegho Sokeng AJ (2017). Targeting signal transducers and activators of transcription (STAT) in human cancer by dietary polyphenolic antioxidants. Biochimie.

[19] Ghadernezhad N, Khalaj L, Pazoki-Toroudi H, Mirmasoumi M, Ashabi G (2016). Metformin pretreatment enhanced learning and memory in cerebral forebrain ischaemia: the role of the AMPK/BDNF/P70SK signalling pathway. Pharm Biol.

[20] Gorjipour F, Dehaki MG, Totonchi Z, Hajimiresmaiel SJ, Azarfarin R, Pazoki-Toroudi H (2017). Inflammatory cytokine response and cardiac troponin I changes in cardiopulmonary bypass using two cardioplegia solutions; del Nido and modified St Thomas’: a randomized controlled trial. Perfusion.

[21] Javedan G, Shidfar F, Davoodi SH, Ajami M, Gorjipour F, Sureda A (2016). Conjugated linoleic acid rat pretreatment reduces renal damage in ischemia/reperfusion injury: Unraveling antiapoptotic mechanisms and regulation of phosphorylated mammalian target of rapamycin. Mol Nutr Food Res.

[22] Pazoki-Toroudi H, Amani H, Ajami M, Nabavi SF, Braidy N, Kasi PD (2016). Targeting mTOR signaling by polyphenols: A new therapeutic target for ageing. Ageing Res Rev.

[23] Pazoki-Toroudi HR, Ajami M, Habibey R (2010). Pre-medication and renal pre-conditioning: a role for alprazolam, atropine, morphine and promethazine. Fundam Clin Pharmacol.

[24] Pazoki-Toroudi HR, Hesami A, Vahidi S, Sahebjam F, Seifi B, Djahanguiri B (2003). The preventive effect of captopril or enalapril on reperfusion injury of the kidney of rats is independent of angiotensin II AT1 receptors. Fundam Clin Pharmacol.

[25] Alikarami F, Yari F, Amirizadeh N, Nikougoftar M, Jalili MA (2015). The Immunosuppressive Activity of Amniotic Membrane Mesenchymal Stem Cells on T Lymphocytes. Avicenna J Med Biotechnol.

[26] Gheisari Y, Azadmanesh K, Ahmadbeigi N, Nassiri SM, Golestaneh AF, Naderi M (2012). Genetic modification of mesenchymal stem cells to overexpress CXCR4 and CXCR7 does not improve the homing and therapeutic potentials of these cells in experimental acute kidney injury. Stem Cells Dev.

[27] Naldini L, Blomer U, Gallay P, Ory D, Mulligan R, Gage FH (1996). In vivo gene delivery and stable transduction of nondividing cells by a lentiviral vector. Science.

[28] Wu Y, Yin X, Wijaya C, Huang MH, McConnell BK (2011). Acute myocardial infarction in rats. J Vis Exp.

[29] Darbandi Azar A, Tavakoli F, Moladoust H, Zare A, Sadeghpour A (2014). Echocardiographic evaluation of cardiac function in ischemic rats: value of m-mode echocardiography. Res Cardiovasc Med.

[30] Esmaeili R, Sadeghpour A, Darbandi-Azar A, Majidzadeh AK, Vajhi A, Sadeghizadeh M (2017). Echocardiographic assessment of myocardial infarction: comparison of a rat model in two strains. Iran J Vet Res.

[31] Ouyang J, Guzman M, Desoto-Lapaix F, Pincus MR, Wieczorek R (2009). Utility of desmin and a Masson’s trichrome method to detect early acute myocardial infarction in autopsy tissues. Int J Clin Exp Pathol.

[32] Tsuji H, Miyoshi S, Ikegami Y, Hida N, Asada H, Togashi I (2010). Xenografted human amniotic membrane-derived mesenchymal stem cells are immunologically tolerated and transdifferentiated into cardiomyocytes. Circ Res.

[33] Shen X, Pan B, Zhou H, Liu L, Lv T, Zhu J (2017). Differentiation of mesenchymal stem cells into cardiomyocytes is regulated by miRNA-1-2 via WNT signaling pathway. J Biomed Sci.

[34] Hare JM, Fishman JE, Gerstenblith G, DiFede Velazquez DL, Zambrano JP, Suncion VY (2012). Comparison of allogeneic vs autologous bone marrow-derived mesenchymal stem cells delivered by transendocardial injection in patients with ischemic cardiomyopathy: the POSEIDON randomized trial. JAMA.

[35] Shadrin IY, Yoon W, Li L, Shepherd N, Bursac N (2015). Rapid fusion between mesenchymal stem cells and cardiomyocytes yields electrically active, non-contractile hybrid cells. Sci Rep.

